# Sex differences in comorbidities and COVID-19 mortality–Report from the real-world data

**DOI:** 10.3389/fpubh.2022.881660

**Published:** 2022-08-12

**Authors:** Yilin Yoshida, Jia Wang, Yuanhao Zu

**Affiliations:** ^1^Section of Endocrinology and Metabolism, Deming Department of Medicine, Tulane University School of Medicine, New Orleans, LA, United States; ^2^Department of Biostatistics and Data Science, Tulane University School of Public Health and Tropical Medicine, New Orleans, LA, United States

**Keywords:** sex differences, comorbidities, COVID-19 mortality, COVID-19 Research Database, real-world data

## Abstract

**Background:**

The differential effect of comorbidities on COVID-19 severe outcomes by sex has not been fully evaluated.

**Objective:**

To examine the association of major comorbidities and COVID-19 mortality in men and women separately.

**Methods:**

We performed a retrospective cohort analysis using a large electronic health record (EHR) database in the U.S. We included adult patients with a clinical diagnosis of COVID-19 who also had necessary information on demographics and comorbidities from January 1, 2016 to October 31, 2021. We defined comorbidities by the Charlson Comorbidity Index (CCI) using ICD-10 codes at or before the COVID-19 diagnosis. We conducted logistic regressions to compare the risk of death associated with comorbidities stratifying by sex.

**Results:**

A total of 121,342 patients were included in the final analysis. We found significant sex differences in the association between comorbidities and COVID-19 death. Specifically, moderate/severe liver disease, dementia, metastatic solid tumor, and heart failure and the increased number of comorbidities appeared to confer a greater magnitude of mortality risk in women compared to men.

**Conclusions:**

Our study suggests sex differences in the effect of comorbidities on COVID-19 mortality and highlights the importance of implementing sex-specific preventive or treatment approaches in patients with COVID-19.

## Background

The coronavirus disease 2019 (COVID-19) has caused a worldwide pandemic. Emerging evidence from clinical and epidemiological studies has suggested that COVID-19 patients with comorbidities are more likely to die from COVID-19 (1–3). Yet, which specific comorbidities predict higher mortality is unclear. Additionally, our understanding of the differential impact of comorbidities by sex is hindered by sex-aggregated analyses in the COVID-19 literature. Menwith COVID-19 appeared to have a higher prevalence of the overall comorbidities, with a higher burden of hypertension, coronary artery disease, ischemic heart disease, or chronic obstructive pulmonary disease highlighted in the major case series since the pandemic, whereas female patients were more likely to have dementia or obesity at the diagnosis of COVID-19 (1, 4–7). The prevalence and impact of diabetes, liver disease, or chronic kidney disease in COVID-19 varied by sex and region (1–4, 8–10). These findings suggest a heterogeneous effect of comorbidities on COVID-19 severe outcomes in mens vs. women but remain to be further clarified. Performing sex-disaggregated analysis in COVID-19 is important as it allows a risk stratification that enables clinicians to use a sex-specific treatment for patients. It also supports sex-tailored public health policies to ensure the most vulnerable group is safe and furthers the effort to keep those who may need intense care from hospitalization. The objective of this study is to leverage a large electronic medical record (EHR) database in the U.S. to investigate the impact of comorbidities on COVID-19 mortality in male and female patients separately.

## Methods

### Study design

This is a retrospective cohort study of adults 18 years or older with a COVID-19 diagnosis (ICD-10 code: U07.1.) from the HealthJump EHR, including diagnosis, procedures, labs, vitals, medications, and histories through the COVID-19 Research Database (11). The COVID-19 Research Database is a pro-bono, public-private consortium composed of institutions that contribute de-identified data of patients with COVID-19. HealthJump, a partnering institution, receives and anonymizes the origin of EHR data from over 500 healthcare organizations in the U.S. We included patients with necessary information on age, sex, race, state of residence, and comorbidities from January 1, 2016 (the earliest available data from the database) to October 31, 2021. We defined comorbidities by the Charlson Comorbidity Index (CCI) using ICD-10 codes at or before the COVID-19 diagnosis (Supplementary Table 1). CCI components include myocardial infarction, congestive heart failure, peripheral vascular disease, cerebrovascular disease, dementia, chronic pulmonary disease, rheumatic disease, peptic ulcer disease, liver disease, diabetes, hemiplegia or paraplegia, renal disease, malignancy, metastatic solid tumor, and AIDS/HIV. Each component was assigned a score when computing the weighted CCI (12). In particular, diabetes with complications, hemiplegia/paraplegia, renal disease, and malignancies are assigned a score of 2; moderate/severe liver disease is assigned a score of 3; metastatic solid tumor and AIDS/HIV are assigned a score of 6; the remaining comorbidities are assigned a score of 1. Our outcome, all-cause mortality, was based on obituary data sourced from online newspapers, funeral homes, online memorials, direct submissions, and more. A death rate of each comorbidity overall and by sex was presented in Supplementary Table 2.

### Analysis

We used the Chi-square test to compare frequencies of comorbidities by sex. We then applied logistic regression for the association between each comorbidity (i.e., CCI component) or the number of comorbidities (i.e., CCI ≥ 2, CCI ≥ 3, CCI ≥ 4, and CCI ≥ 5) and mortality in the overall sample followed by a sex-stratified analysis. A trend analysis was conducted for the increased number of comorbidities for the overall and sex-specific analysis. In the multivariable model, we adjusted for age, race, and state of residence. We also performed the same analysis in non-Hispanic white and non-Hispanic black sample, separately. All model-based results were presented with 95% confidence intervals. We used SAS version 9.4 (SAS Institute) for the analysis.

## Results

We included 121,342 patients with complete information on age, race, state, and comorbidities in our analysis. The mean age of our sample was 48 years old, with male patients slightly older than female patients (48 vs. 47 years old). More than half of the sample was non-Hispanic whites (whites) followed by 17.2% non-Hispanic blacks (blacks), and another 2.1% from other ethnic minorities. There was a higher percentage of black patients in women (19% and 15% in women and men, respectively). The most prevalent comorbidities in our sample were chronic pulmonary disease (19%), diabetes without complication (13%), and renal disease (7.7%). The cardiovascular complications including myocardial infarction, congestive heart failure, and peripheral vascular disease, were around 5%. There was a significant sex difference in the prevalence of the majority of the CCI components, with male patients having a higher prevalence of myocardial infarction, congestive heart failure, peripheral vascular disease, diabetes, renal disease, malignancy, and AIDS/HIV, whereas female patients had a higher prevalence of dementia, chronic pulmonary disease, and rheumatic disease (Table 1).

**Table 1:** Characteristics of COVID-19 patients by sex.

	**All (*n* = 121,342)**	**Women (*n* = 70,668)**	**Men (*n* = 50,674)**	***P*-value**
Age (mean, SD)	47.75 (13.82)	47.28 (13.89)	48.41 (13.70)	<0.0001
**Race (** ***n*** **, %)**			
White	97,826 (80.62)	55,620 (78.71)	42,206 (83.29)	<0.0001
Black	20,918 (17.24)	13,520 (19.13)	7,398 (14.60)	
Others	2,598 (2.14)	1,528 (2.16)	1,070 (2.11)	
**Top 5 states (** ***n*** **, %)**			
North Carolina	23,820 (19.63)	13,921 (19.70)	9,899 (19.53)	<0.0001
California	16,914 (13.94)	10,149 (14.36)	6,765 (13.35)	
Louisiana	14,420 (11.88)	8,649 (12.24)	5,771 (11.39)	
Arkansas	9,723 (8.01)	5,776 (8.17)	3,947 (7.79)	
Mississippi	6,328 (5.22)	3,843 (5.44)	2,485 (4.90)	
Myocardial infarction (*n*, %)	1,442 (1.19)	649 (0.92)	793 (1.56)	<0.0001
Congestive heart failure (*n*, %)	5,529 (4.56)	2,870 (4.06)	2,659 (5.25)	<0.0001
Peripheral vascular disease (*n*, %)	5,713 (4.71)	2,927 (4.14)	2,786 (5.50)	<0.0001
Cerebrovascular disease (*n*, %)	5,264 (4.34)	2,966 (4.20)	2,298 (4.53)	0.0044
Dementia (*n*, %)	2,237 (1.84)	1,390 (1.97)	847 (1.67)	0.0002
Chronic pulmonary disease (*n*, %)	22,757 (18.75)	14,754 (20.88)	8,003 (15.79)	<0.0001
Rheumatic disease (*n*, %)	3,014 (2.48)	2,377 (3.36)	637 (1.26)	<0.0001
Peptic ulcer disease (*n*, %)	996 (0.82)	611 (0.86)	385 (0.76)	0.0459
Mild liver disease (*n*, %)	5,193 (4.28)	2,991 (4.23)	2,202 (4.35)	0.3377
Moderate or severe liver disease (*n*, %)	255 (0.21)	125 (0.18)	130 (0.26)	0.0028
Liver disease (mild, moderate or severe), (*n*, %)	5239 (4.32)	3012 (4.26)	2,227 (4.39)	0.2625
Diabetes without chronic complication (*n*, %)	15,421 (12.71)	8,556 (12.11)	6,865 (13.55)	<0.0001
Diabetes with chronic complication (*n*, %)	9,561 (7.88)	5,142 (7.28)	4,419 (8.72)	<0.0001
Diabetes (with or without complication), (*n*, %)	24,982 (20.59)	13,698 (19.38)	11,284 (22.27)	<0.0001
Hemiplegia or paraplegia (*n*, %)	559 (0.46)	296 (0.42)	263 (0.52)	0.0111
Renal disease (*n*, %)	9,395 (7.74)	5,092 (7.21)	4,303 (8.49)	<0.0001
Any malignancy, including lymphoma and leukemia, except malignant neoplasm of skin (*n*, %)	4,649 (3.83)	2,341 (3.31)	2,308 (4.55)	<0.0001
Metastatic solid tumor (*n*, %)	366 (0.30)	199 (0.28)	167 (0.33)	0.1330
Cancer (any malignancy or metastatic solid tumor), (*n*, %)	4,740 (3.91)	2,391 (3.38)	2,349 (4.64)	<0.0001
AIDS/HIV (*n*, %)	383 (0.32)	139 (0.20)	244 (0.48)	<0.0001
CCI mean score (SD)	1.00 (1.63)	0.97 (1.57)	1.04 (1.72)	<0.0001
CCI ≥ 2 (*n*, %)	28,389 (23.40)	15,970 (22.60)	12,419 (24.51)	<0.0001
CCI ≥ 3 (*n*, %)	16,121 (13.29)	8,856 (12.53)	7,265 (14.34)	<0.0001
CCI ≥ 4 (*n*, %)	10,002 (8.24)	5,325 (7.54)	4,677 (9.23)	<0.0001
CCI ≥ 5 (*n*, %)	5,944 (4.90)	3,122 (4.42)	2,822 (5.57)	<0.0001
Death (*n*, %)	2,901 (2.39)	1,420 (2.01)	1,481 (2.92)	<0.0001

**Figure 1:**
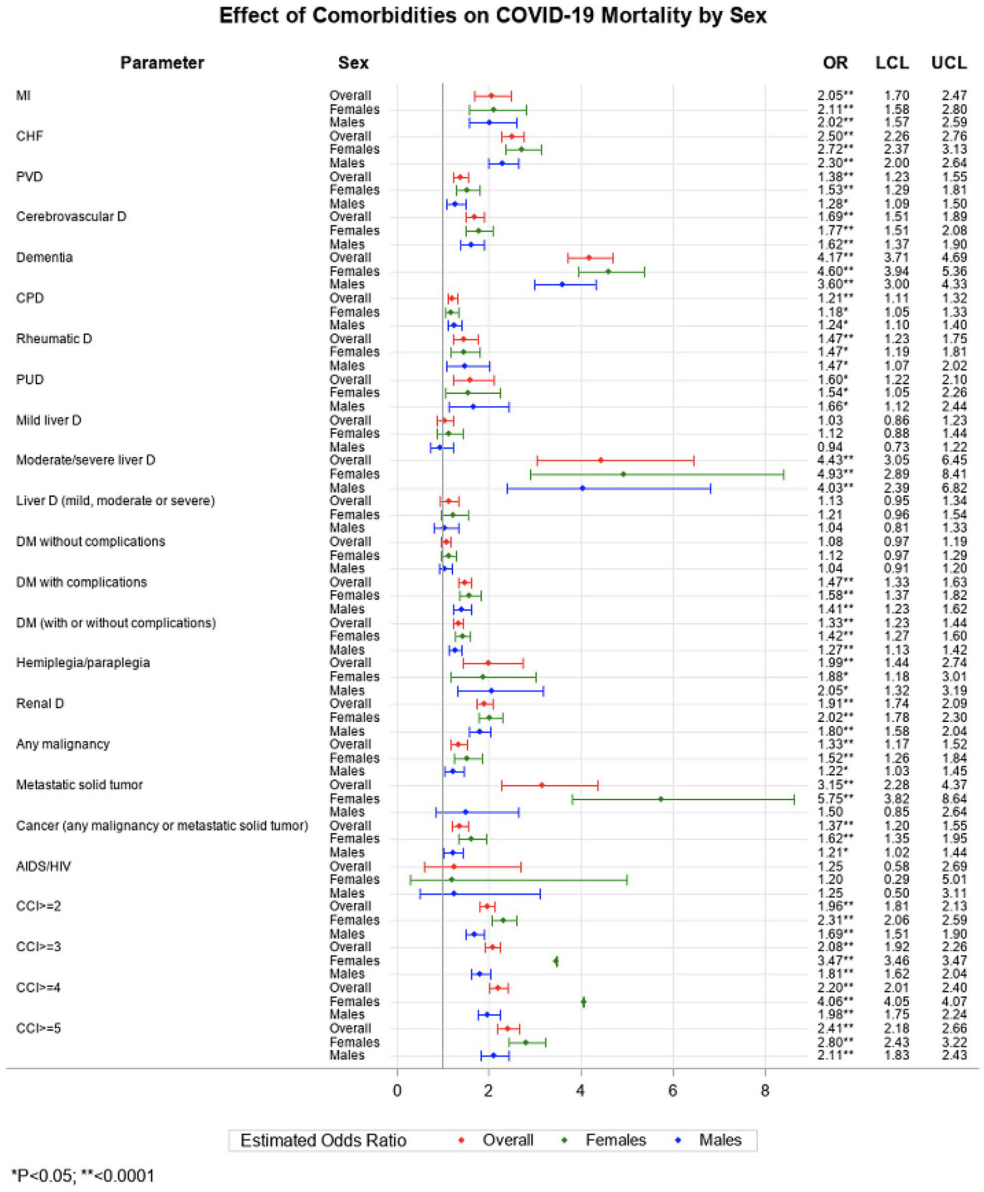
Odds ratio of comorbidities and mortality in female and male patients with COVID-19.

In the fully adjusted logistic regressions (Figure 1), we found most of the individual CCI components were associated with an elevated risk of death in COVID-19 patients. Notably, moderate/severe liver disease [odds ratio (OR) 4.4, 95% confidence interval (CI) 3.1–6.5], dementia (4.2, 3.7–4.7), metastatic solid tumor (3.2, 2.3–4.4), heart failure (2.5, 2.3-2.8), and myocardial infarction (2.1, 1.7–2.5) were associated more than 2-fold higher odds of death in the overall sample. In female patients, the top impactful comorbidities on COVID-19 mortality were metastatic solid tumor (5.8, 3.8–8.6), moderate/severe liver disease (4.9, 2.9–8.4), dementia (4.6, 3.9–5.4), heart failure (2.7, 2.4–3.1), myocardial infarction (2.1, 1.6–2.8), and renal disease (2.0, 1.8–2.3), while in male patients, the top fetal comorbidities were moderate/severe liver disease (4.0, 2.4–6.8), dementia (3.6, 3.0–4.3), heart failure (2.3, 2.0–2.6), and hemiplegia or paraplegia (2.1, 1.3–3.2). The increased number of CCI components was associated with an increased risk of death in men and women (*P*-trend < 0.05, not shown in the figure). The incremental risk of death associated with the rising number of comorbidities was stronger in women than in men.

In non-Hispanic white (white) patients with COVID-19, the most impactful comorbidities in mortality were myocardial infarction (7.51, 7.48–7.54), dementia (4.2, 3.7–4.8), cerebrovascular disease (2.84, 2.83–2.85), heart failure (2.7, 2.4-2.9), and renal disease (2.426, 2.42–2.43) (Supplementary Figure 1). In non-Hispanic black (black) patients, top comorbidities associated with COVID-19 mortality were dementia (3.6, 2.7–4.7), cerebrovascular disease (2.1, 1.7–2.7), and renal disease (1.98, 1.64–2.39) (Supplementary Figure 2). In both white and black patients, the impact of comorbidities on mortality was more pronounced in women compared to men (Supplementary Figures 1, 2).

## Discussion

From this large EHR analysis, we confirmed a significant role of comorbidities in COVID-19 mortality. The risk of death significantly increased with the rising number of comorbidities. Of note, moderate to severe liver disease, dementia, metastatic solid tumor, congestive heart failure, and myocardial infarction were the top five fatal commodities in COVID-19 patients. Importantly, our study suggests sex differences in the effect of comorbidities on COVID-19 mortality. In contrast to prior studies that suggested the higher COVID-19 mortality may be contributed by the higher burden of comorbidities in men (5, 6), our analysis demonstrated that the impact of comorbidities on COVID-19 death is more pronounced in women. The magnitude of risk of death associated with comorbidities was larger in women across CCI categories (CCI ≥ 2, CCI ≥ 3, CCI ≥ 4, CCI ≥ 5) and we have observed a stronger incremental change in compared to msen. In addition, the risk of death associated with top comorbidities including moderate to severe liver disease, dementia, metastatic solid tumor, and congestive heart failure was significantly greater in women compared to men (*p* interaction < 0.05). Our findings regarding a more adverse effect of dementia and heart failure in women were consistent with a prior report from Italy (7), underscoring the critical role of these two conditions in predicting COVID-19 death, particularly in women. No study to our knowledge has reported the sex differential effect of liver disease and tumor on COVID-19 death.

Due to the data unavailability, our study included limited sociodemographic, health care access/use, and clinical information, therefore, our analysis is subject to residual confounding. Only all-cause mortality was available as the outcome. Since HealthJump contains mostly outpatient information, we were not able to examine disease severity or hospitalization. The vaccination status was also not available in the database. We were not able to define gender, including a collection of gender-related variables, such as identity, role, or relations. Further, we used a conservative definition for COVID-19 (ICD-10 code), which potentially excluded COVID-19 positive cases who did not acquire a clinical diagnosis. Further, the observational nature of our study precludes causal inference. Despite the limitations, the large EHR data supported sufficient power for a sex-stratified analysis and highlighted the markable sex differences in comorbidities and COVID-19 death in the U.S. The significant impact of comorbidities in female patients with COVID-19 may be underappreciated as previous reports stressed the disproportionate death rate in male patients that are related to their underlying comorbid conditions. Our findings underscore the importance of implementing sex-specific preventive or treatment approaches in the context of COVID-19. More sex-disaggregated research is warranted for understanding the risk factors of poor outcomes and health inequities and for guiding the COVID-19 management.

## Data availability statement

The data analyzed in this study is subject to the following licenses/restrictions: the data that support the findings of this study are available from Healthjump database and the COVID-19 Research Database consortium, but restrictions apply to the availability of these data, which were used under license for the current study, and so are not publicly available. Data are however available from the authors upon reasonable request and with permission of Healthjump database and the COVID-19 Research Database consortium. Requests to access these datasets should be directed to https://covid19researchdatabase.org.

## Author contributions

YY designed the study and supervised all stages of the study, including data acquisition, analysis, and manuscript writing. JW took part in the analysis and generated the table/figure. YZ provided technical support to the analysis. All authors reviewed and approved the final version of the manuscript.

## Funding

This project was supported by the COVID-19 Research Accelerator program (CORONAVIRSUHUB-D-21) and a grant (NIH K12HD043451) from the Eunice Kennedy Shriver National Institute of Child Health and Human Development of the Building Interdisciplinary Research Careers in Women's Health (BIRCWH) Scholar. This project was also supported in part by U54 GM104940 from the National Institute of General Medical Sciences of the National Institutes of Health, which funds the Louisiana Clinical and Translational Science Center.

## Conflict of interest

The authors declare that the research was conducted in the absence of any commercial or financial relationships that could be construed as a potential conflict of interest.

## Publisher's note

All claims expressed in this article are solely those of the authors and do not necessarily represent those of their affiliated organizations, or those of the publisher, the editors and the reviewers. Any product that may be evaluated in this article, or claim that may be made by its manufacturer, is not guaranteed or endorsed by the publisher.

## Author disclaimer

The content is solely the responsibility of the authors and does not necessarily represent the official views of the National Institutes of Health.

## References

[1] Ejaz H Alsrhani A Zafar A Javed H Junaid K Abdalla AE. COVID-19 and comorbidities: deleterious impact on infected patients. J Infect Public Health. (2020) 13:1833–9. 10.1016/j.jiph.2020.07.014 32788073 PMC7402107

[2] Li J He X Yuan Y Zhang W Li X Zhang Y. Meta-analysis investigating the relationship between clinical features, outcomes, and severity of severe acute respiratory syndrome coronavirus 2 (SARS-CoV-2) pneumonia. Am J Infect Control. (2021) 49:82–9. 10.1016/j.ajic.2020.06.008 32540370 PMC7292004

[3] Singh AK Gillies CL Singh R Singh A Chudasama Y Coles B. Prevalence of co-morbidities and their association with mortality in patients with COVID-19: a systematic review and meta-analysis. Diabetes Obes Metab. (2020) 22:1915–24. 10.1111/dom.14124 32573903 PMC7361304

[4] Naaraayan A Nimkar A Pant S Hasan A Durdevic M Elenius H. Sex disparity in the effect of obesity in hospitalized COVID-19 patients: a retrospective cohort study from the New York city metropolitan area. Cureus. (2021) 13:e15235. 10.7759/cureus.15235 34178545 PMC8223951

[5] Nguyen NT Chinn J De Ferrante M Kirby KA Hohmann SF Amin A. Male gender is a predictor of higher mortality in hospitalized adults with COVID-19. PLoS ONE. (2021) 16:e0254066. 10.1371/journal.pone.0254066 34242273 PMC8270145

[6] Suleyman G Fadel RA Malette KM. Clinical characteristics and morbidity associated with coronavirus disease 2019 in a series of patients in metropolitan detroit. JAMA Netw Open. (2020) 3:e2012270. 10.1001/jamanetworkopen.2020.12270 32543702 PMC7298606

[7] Raparelli V Palmieri L Canevelli M Pricci F BUnim B Noce CL. Sex differences in clinical phenotype and transitions of care among individuals dying of COVID-19 in Italy. Biol Sex Differ. (2020) 11:57. 10.1186/s13293-020-00334-3 33066823 PMC7562690

[8] Cheng R Liu C Yang J Yuanqi Yang 1 Renzheng Chen 1 Ding X. Sex differences in the incidence and risk factors of myocardial injury in COVID-19 patients: a retrospective cohort study. Front Physiol. (2021) 12:632123. 10.3389/fphys.2021.632123 33664674 PMC7920972

[9] Yoshida Y BM Gillet SA Wilson SM Ahmed SJ Tirumalasetty S Zu Y. Clinical characteristics and outcomes in women and men hospitalized for coronavirus disease-2019 in New Orleans. Biol Sex Differ. (2021). 10.1186/s13293-021-00359-2 33546750 PMC7863061

[10] Tramunt B Smati S Coudol S Wargny M Pichelin M Guyomarch B. Sex disparities in COVID-19 outcomes of inpatients with diabetes: insights from the CORONADO study. Eur J Endocrinol. (2021) 185:299–311. 10.1530/EJE-21-0068 34085949 PMC9494335

[11] *COVID-19 Research Database*. Available online at: https://covid19researchdatabase.org/ (accessed October 2021).

[12] Quan H Li B Couris CM Fushimi K Graham P Hider P. Updating and validating the Charlson comorbidity index and score for risk adjustment in hospital discharge abstracts using data from 6 countries. Am J Epidemiol. (2011) 173:676–82. 10.1093/aje/kwq433 21330339

