# Supplementary material for: Sex differences in comorbidities and COVID-19 mortality–Report from the real-world data
Source: Front Public Health. 2022 Aug 12;10:881660. doi: 10.3389/fpubh.2022.881660 (PMC9412184; doi:10.3389/fpubh.2022.881660)
Supplement: Supplementary file 2 [file Table_2.docx]

**Supplemental Table 2. All-cause mortality by comorbidity and sex**

|  | All (n=2901) | Females (n=1420) | Males (n=1481) |
| --- | --- | --- | --- |
| **MI** | 134 (0.09) | 57 (0.09) | 77 (0.097) |
| **CHF** | 606 (0.11) | 297 (0.10) | 309 (0.11) |
| **PVD** | 369 (0.06) | 172 (0.06) | 197 (0.07) |
| **Cerebrovascular D** | 405 (0.08) | 204 (0.07) | 201 (0.09) |
| **Dementia** | 441 (0.2) | 265 (0.19) | 176 (0.2) |
| **CPD** | 793 (0.03) | 415 (0.03) | 378 (0.05) |
| **Rheumatic D** | 147 (0.05) | 102 (0.04) | 45 (0.07) |
| **Peptic ulcer disease** | 60 (0.06) | 30 (0.05) | 30 (0.07) |
| **Mild liver disease** | 137 (0.03) | 71 (0.02) | 66 (0.03) |
| **Moderate or severe liver disease** | 36 (0.14) | 18 (0.14) | 18 (0.14) |
| **Liver disease (mild, moderate or severe)** | 150 (0.03) | 77 (0.03) | 73 (0.03) |
| **Diabetes without chronic complication** | 526 (0.03) | 246 (0.03) | 280 (0.03) |
| **Diabetes with chronic complication** | 550 (0.06) | 268 (0.05) | 282 (0.06) |
| **Diabetes (with or without complication)** | 1076 (0.04) | 514 (0.04) | 562 (0.04) |
| **Hemiplegia or paraplegia** | 44 (0.08) | 20 (0.07) | 24 (0.09) |
| **Renal disease** | 777 (0.08) | 392 (0.08) | 385 (0.08) |
| **Any malignancy, including lymphoma and leukemia, except malignant neoplasm of skin** | 294 (0.06) | 129 (0.06) | 165 (0.07) |
| **Metastatic solid tumor** | 46 (0.13) | 32 (0.16) | 14 (0.08) |
| **Cancer (any malignancy or solid tumor)** | 306 (0.06) | 139 (0.06) | 167 (0.06) |
| **AIDS/HIV** | 7 (0.02) | 2 (0.014) | 5 (0.0.02) |
