# Supplementary figures and images for: Sex differences in comorbidities and COVID-19 mortality–Report from the real-world data
Source: Front Public Health. 2022 Aug 12;10:881660. doi: 10.3389/fpubh.2022.881660 (PMC9412184; doi:10.3389/fpubh.2022.881660)

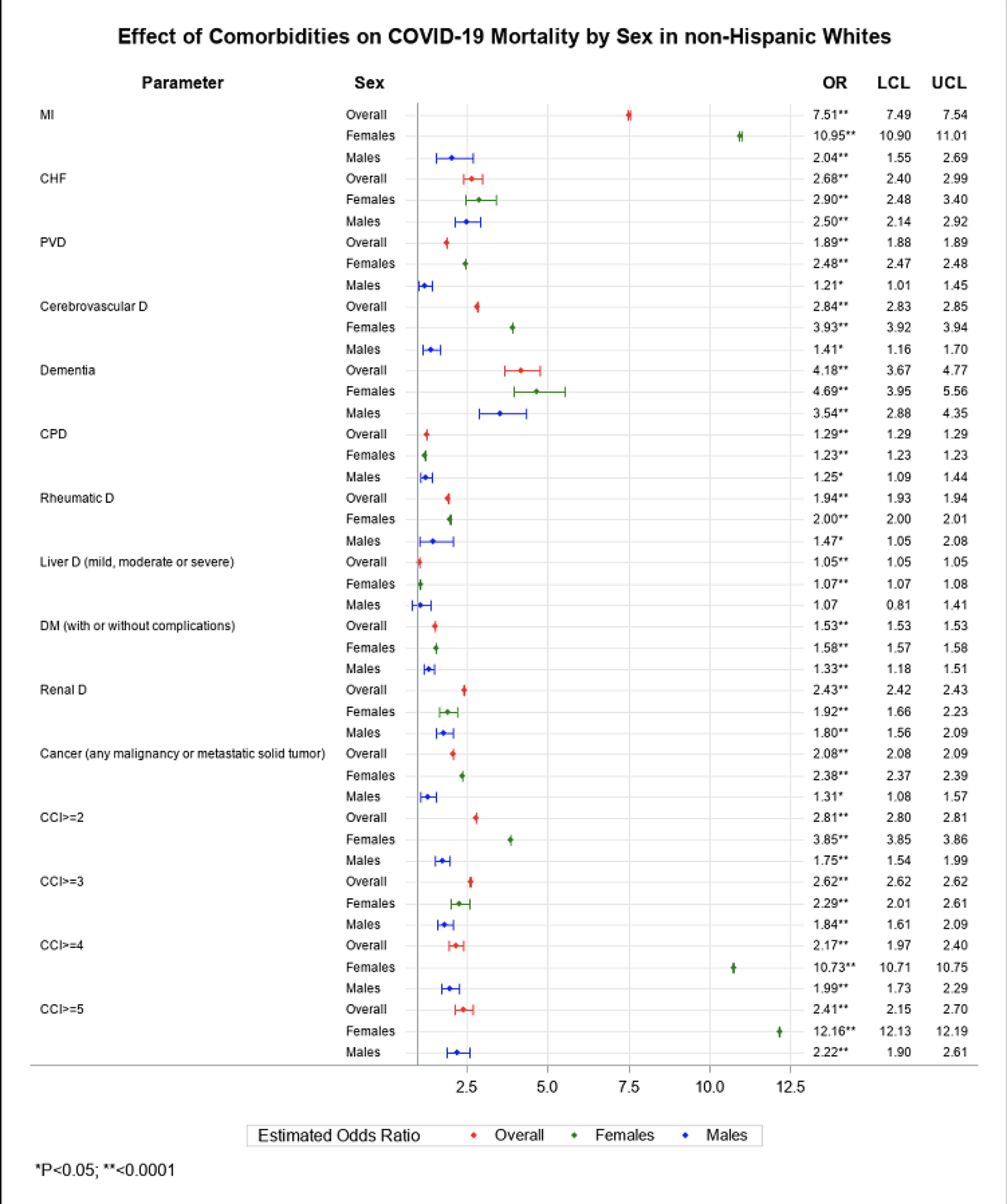

Supplement: Supplementary file 3 [file Image_1.JPEG]

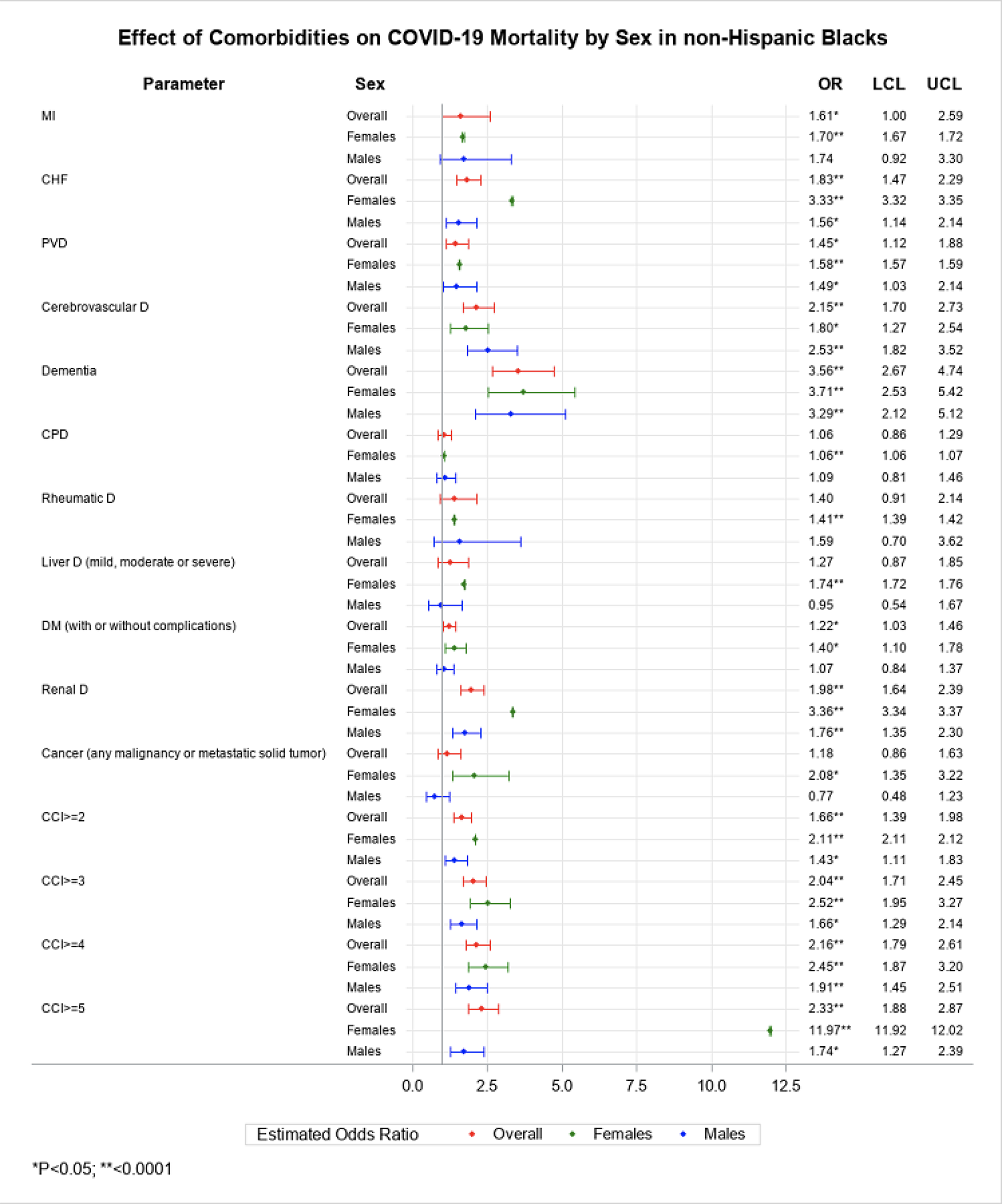

Supplement: Supplementary file 4 [file Image_2.JPEG]
